# Daylight and Electric Lighting in Primary and Secondary School Classrooms in the UK—An Observational Study

**DOI:** 10.3390/ijerph21070942

**Published:** 2024-07-19

**Authors:** Luke L. A. Price, Annegret Dahlmann-Noor, Marina Khazova

**Affiliations:** 1 UK Health Security Agency, Didcot OX11 0RQ, UK; marina.khazova@ukhsa.gov.uk; 2 Moorfields Eye Hospital NHS Foundation Trust, London EC1V 2PD, UK; annegret.dahlmann-noor@nhs.net; 3 Institute of Ophthalmology, University College London, London WC1E 6BT, UK

**Keywords:** daylight, lighting, children, classroom, myopia, light logging, illuminance, spectrum, melanopic EDI, flicker

## Abstract

Only a few recent studies report direct assessment or monitoring of light levels in the indoor learning environment, and no consensus exists on minimum exposures for children’s health. For instance, myopia is a common progressive condition, with genetic and environmental risk factors. Reduced daylight exposure, electric lighting changes, increased near-work for school children, greater academic focus, and use of display screens and white boards may have important detrimental influences. Published assessment methods had varied limitations, such as incomplete compliance from participants wearing light loggers for extended periods. Climate-Based Daylight Modelling is encouraged in UK school design, but design approaches are impractical for post-occupancy assessments of pre-existing classrooms or ad hoc modifications. In this study, we investigated the potential for direct assessment and monitoring of classroom daylight and lighting measurements. Combined with objective assessments of outdoor exposures and class time use, the classroom data could inform design and light exposure interventions to reduce the various health impacts of inadequate daylight exposure. The relevant environmental measure for myopia depends on the hypothesized mechanism, so the illuminance, spectral distribution, and temporal light modulation from the electric lighting was also assessed.

## 1. Introduction

Children’s exposure to outdoor light has reduced dramatically over the past 50 years [1]. Suboptimal light exposure is being implicated in adverse health outcomes, and for myopia, in particular, evidence has been growing that a reduction in outdoor light exposure is associated with an earlier onset of myopia [2,3,4]. Most research on this topic has, to date, been carried out in East Asia and Australia [5,6,7,8]. However, over the past 50 years, the prevalence of myopia in children in the UK has doubled [9], and the pace may be accelerating since the COVID19-pandemic lockdowns [10]. Children from Asian and Black families have a 9× and 3× higher risk than White children, respectively, of developing myopia [11].

It is estimated that now nearly 50% of young adults in Europe have myopia [12]. Myopia can lead to blindness [13]. Whilst the risk increases exponentially with the increasing degree of myopia, sight-threatening complications also occur at moderate levels [14]. Sight-impairment statistics predict that the greatest rise in blindness in the UK will affect people from ethnic minority groups: by 2050, the share of White people with blindness is predicted to fall from 94.9% to 88.5%, whilst that of Black people is projected to increase from 1.0% to 1.8%, Asians from 3.0% to 6.4%, and other ethnicities from 1.1% to 3.3% [15].

Myopia onset can be delayed by increasing time spent outdoors [16]; this reduces the final degree of myopia, which is intimately linked with blinding complications in adulthood [14]. Sustained visual attention on objects in the near field is considered another important risk factor [17], partly resulting from the prolonged contraction of the ciliary muscle that increases the lens’s focusing power. The child’s environment during the early years of life, therefore, plays a key role in promoting or delaying myopia onset.

A national birth cohort study in England, Children of the 2020s, recently reported that already at the age of 9 months there are substantial differences in children’s exposure to these two main modifiable risk factors: only 21% and 31% of primary caregivers of Black/Black British and Mixed/Other ethnicity, respectively, can take their child to an outdoor/green/natural space every day, compared to 41% of White primary caregivers [18]. Conversely, daily exposure to digital screens is highest in Black/Black British babies, at nearly 50 min/day, compared with 30 min/day in babies from all other ethnic groups [18].

### 1.1. Assessing Lighting Quality

It may be possible to mitigate the consequences of reduced exposure to light outdoors, at least in part, with improved indoor lighting. Daylight is the most appropriate primary source of lighting for classrooms, and guidance exists on design through applying Climate-Based Daylight Modelling (CBDM) [19]. For existing classrooms, data may not exist to enable CBDM, and it may be time-consuming to collect manually. CBDM uses historic environmental data to simulate daylight availability within architectural spaces and consider its statistical distribution over long time series [19]. This or similar modelling paradigms can inform architectural and lighting design of new school buildings and planned modifications, and when applied to existing and occupied classrooms, may support post-occupancy exposure assessments. In contrast, monitoring daylight captures actual conditions, with information that is potentially more dynamic and better related to eye health than the daylight factor.

The main emphasis of indoor electric lighting has often been on providing sufficient illumination of viewed objects and working surfaces for visual purposes, i.e., in the absence of daylight. For eye health and circadian rhythms, exposures in the vertical plane of the eyes should be assessed [20], including the contributions of daylight and electric lighting.

The spectrum for many existing LED lighting products consists of a narrow blue peak at about 445 to 455 nm, directly from the LED component, combined with a broad yellow peak from one or more LED-stimulated phosphors, centred at about 550 to 560 nm. In combination, these provide a white light. This light excludes the short and long wavelength regions of the visible spectrum. Compared to daylight, it is also lower in light around 490 nm, which sets the central regulator of circadian rhythm timing [21].

Temporal light modulation (TLM) at 100 Hz and above can often result from LED lighting run on the alternating-current UK main power supply (line frequency of 50 Hz). The term flicker has been used to indicate directly visible TLM at up to ~80 Hz, which is less common in mains-driven lighting [22,23,24]. Installed lighting using rectification and pulse-width modulation above approximately 90 Hz can be associated with physiological responses based on saccadic eye movement during high-contrast activities such as reading, even though flicker is not visible to observers in laboratory studies [25,26]. Typical LED lighting waveforms have been shown in several publications elsewhere, including a health-related review of LED lighting solutions in the UK [27].

### 1.2. Objectives

This study’s aim is to explore how objective light exposure factors related to health can be collected and quantified in school classrooms:

1. To monitor vertical daylight illuminance entering the classroom and arriving at the back of the classroom during school hours, to assess daylight penetration and daylight autonomy against vertical illuminance targets;
2. To collect vertical grid eye height illuminance—snapshots during school hours, supported by monitoring data, with lights on and off—as descriptive data at potential exposure positions;
3. To collect spectral distribution data for electric lighting to determine the Correlated Colour Temperature (CCT) and melanopic Daylight Efficacy Ratio (m-DER) [28], as measures describing the non-visual stimulus for a given illuminance;
4. To collect TLM data for electric lighting to determine its flicker frequency and physiological percent flicker (PPF) [24], as an assessment against international consensus recommendations [25], including post-measurement validation of signal-to-noise being sufficient to support the assessments.

## 2. Materials and Methods

Schools in Bedford, UK, were approached about taking part in the study. Two primary and two secondary schools agreed, each of which contributed four daylit classrooms. All schools had one or two floors of classrooms throughout, and in the latter case, the classrooms were selected to include roughly equal numbers of ground and first-floor rooms, with different aspects within floors. One school contributed a fifth interior classroom, where the windows onto other rooms admitted no appreciable daylight; this room was excluded from daylight assessments.

### 2.1. Measurements

The schools were visited over the course of two days, and installed equipment was collected after at least six further complete days (6 to 11 April 2023), before the end of the holiday period, with the chairs in each classroom placed on the floor throughout.

Two light loggers (Modified ActTrust model number AT0503LF, Condor Instruments, Sao Paolo, Brazil) were placed at the same height: one in the centre of the main aspect window of the room facing outwards and the second on the opposite facing wall, i.e., facing in the same direction. The loggers were set to collect light data at one-minute intervals; their suitability for illuminance and melanopic equivalent daylight illuminance (m-EDI) has been previously confirmed [29].

A hand-held illuminance meter with remote head (T-10A, Konika-Minolta, Tokyo, Japan) was used to measure vertical illuminances. Values were sense-checked and noted by a second researcher. The measurements were taken alongside the light loggers and in four directions at selected grid points with lights on and off. Five grid points were selected in a cross shape, or quincunx, comprised of the quartiles on the room diagonals. The combined linearity, spectral sensitivity, and cosine mismatch of the illuminance meter was within 10%, whilst most relative errors between measurements should be substantially lower and display precision errors below 1%.

The grid-point measurements were taken at a height of 1.0 m to represent the position of the eyes of an average-height seated child, and light loggers were placed at 1.5 m for daylight penetration above most obstructions, including during the monitoring period.

For one luminaire per classroom, the emitted spectrum and temporal modulation were measured. Measurements were not repeated if there were identical luminaires in one or more classrooms. The equipment used, or similar equipment, has been previously reported [27].

Background adjustment and calibration were applied as required to the raw data, and relevant data were selected, checked, and cleaned for analysis. The sampling control method was not sufficient to support detailed sample-based statistical testing, and analysis was, therefore, conducted to summarize baseline characteristics, to explore factors related to variability, and to support exposure assessments against consensus health recommendations.

### 2.2. Assessments

From monitoring data, vertical daylight illuminance at the main glazed aspect and daylight penetration were tested and considered using recommendations for circadian rhythms of day-active adults in terms of a daylight spectrum [20]. These recommendations were expressed using m-EDI measured at eye level, and the daytime target was 250 lx. The monitoring data were investigated to confirm the potential use of fixed loggers to estimate typical classroom exposures over time, including against the 250 lx target and other potential thresholds. The school hours were all assumed to be from 08:30 to 16:29.

**Figure 1:**
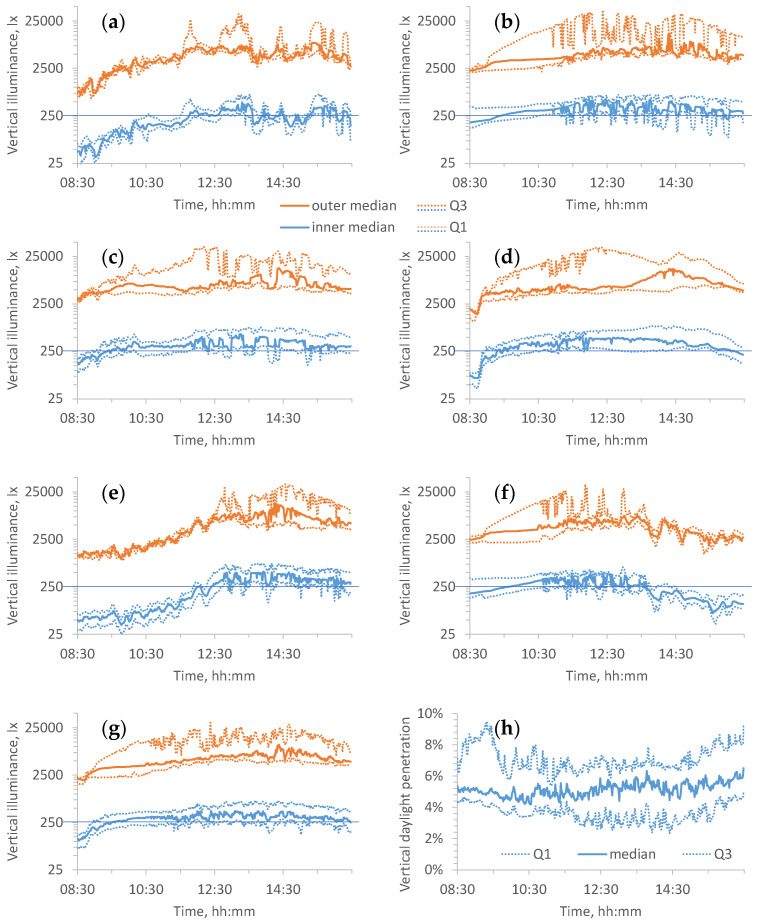
Quartiles between classrooms of vertical daylight illuminance (lx) at the outer and inner sensors at 1.5 m height during “school time” from 08:30 to 16:29 on 6–11 April 2023 (days 3–8). (**a**–**f**) Individual days, with the remaining panels showing the medians for all days combined, (**g**) the quartiles on individual days, for vertical illuminance, and (**h**) daylight penetration. Vertical illuminance of 250 lx at the inner sensor is indicated in (**a**–**g**), representing the recommended eye-level exposure when light is provided by daylight alone [20].

Similarly, the vertical eye height illuminance grid data were also compared with this fixed target, with lights both on and off. We tested electric lighting spectra for CCT and m-DER, the latter being the ratio of m-EDI to illuminance, which, for UK daylight, has been confirmed to be close to 1.0 at most times [21,28]. Due to being widely available, CCT has often been used as a proxy for m-DER [28]. As m-DER is close to 1.0 for most daylight spectra, the m-EDI target corresponds closely to an illuminance target of 250 lx, when the m-DER of an electric light source is used as a correction factor for its contribution.

Lastly, we tested temporal modulation against 2015 international recommendations (Institute of Electrical and Electronics Engineers, IEEE) for avoiding health problems in viewers due to flicker [25]. For modulation with a frequency of 90 Hz and above, temporally smoothed data determines an adjusted percent flicker. This metric is known as physiological percent flicker or PPF [24], with a value of 3% corresponding to the IEEE “low risk limit”, and a value of 1% to the region of “no observable effect limit”.

## 3. Results

Figure 1 shows the quartiles across classrooms of illuminance due to daylight alone on the windows (“outer”) and at the back of the classrooms (“inner”) collected by the loggers over individual days (a–f), for the assumed school hours. (g) shows the medians across six days of the quartiles of both the inner and outer illuminances, i.e., the medians of (a–f), whilst (h) shows the same for the ratio of inner and outer illuminances, representing the daylight penetration to the back of the classrooms for paired data only. A total of 13 loggers were excluded; two did not record complete data, one did not stay in place, and the rest suffered battery failures prior to data retrieval. Hence, nineteen out of thirty-two loggers were included (ten inner and nine outer) for (a–g), with six out of sixteen pairs of loggers for (h), providing 109,440 measurements or 912 h of data, or 288 h for pairs.

**Figure 2:**
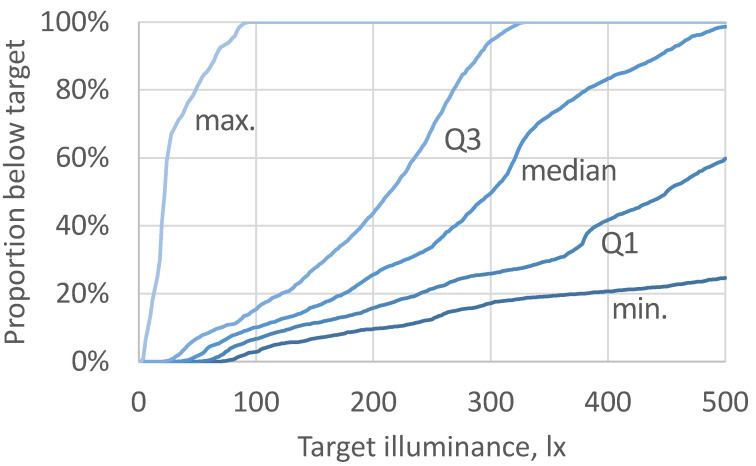
Proportion of logged school time including breaks where the maximum, Q3, median, Q1, and minimum daylight illuminance, measured from all inner loggers in classrooms, reached different illuminance targets during typical school hours on days 3–8.

The maximum, Q3, median, Q1, and minimum of all inner illuminance loggers fell below the 250 lx target 12%, 21%, 34%, 69%, and 100% of the time, respectively. Halving the target, these proportions became 5%, 9%, 13%, 20%, and 100%. Figure 2 generalizes this analysis to any target illuminance between 0 and 500 lx.

**Figure 3:**
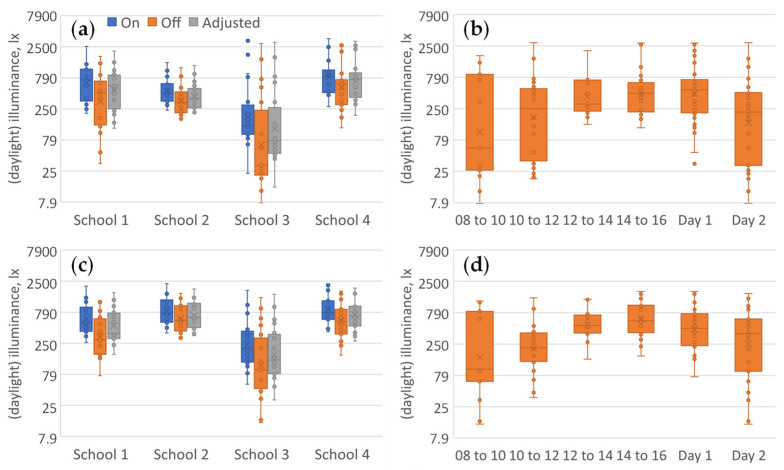
Box and whisker plots of vertical grid illuminance (lx) at seated eye-height (log scales, daylit rooms only). (**a**) Combined spot measurements facing forward by school for lights on (left series, blue), lights off (middle series, orange), and the adjusted equivalent daylight illuminance with lights on (right series, grey, i.e., allowing for m-DER). (**b**) The combined schools’ forward facing data split by time-of-day and by day-of-visit for lights off only. (**c**,**d**) The same analysis for data combined for all four directions of view.

Figure 3 shows classroom grid illuminance at seated eye height grouped by school, time-of-day, and day-of-visit. Visual comparison with Figure 3 shows variations between and within days that parallel the grid illuminance variations (days 1 and 2).

**Figure 4:**
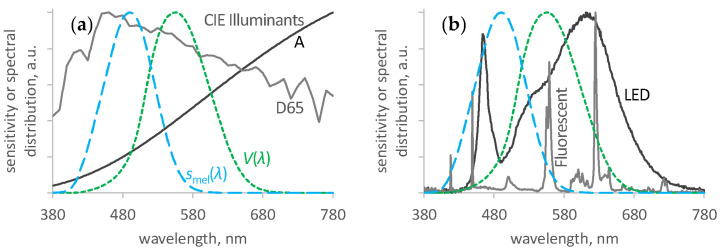
Relative spectral distributions of light sources, including the International Commission on Illumination (CIE) standard visual (photopic, dotted green line, *V*(*λ*)) and non-visual (melanopic, dashed blue line, *s*_mel_(*λ*)) spectral sensitivities [28]. (**a**) CIE illuminant A, typifying phased-out incandescent light (dark grey line, CCT = 2856 K, m-DER = 50%), and CIE daylight illuminant D65, representing indirect daylight (light grey line, CCT = 6500 K, m-DER ≡ 100%) [21]. (**b**) LED lighting in schools 1 and 4 (dark grey line), and fluorescent light in schools 2 and 3 (light grey line); typical examples shown.

The mean CCT for classroom electric lights was 3000 K (s.d. 59 K, range 2900 K to 3100 K) and the mean m-DER was 45% (s.d. 4.2%, range 40% to 50%), see Figure 4. The Pearson correlation coefficient was 29%, indicating a weak relationship between observed CCT and m-DER. The LED spectra were consistent with the description in the introduction, including the spectrum shown in Figure 4b.

**Figure 5:**
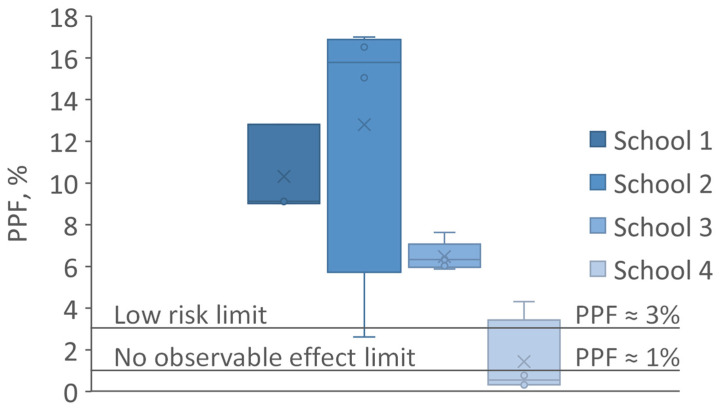
Box and whisker TLM plots (*n* = 3, 4, 5, and 4 for schools 1–4, respectively) showing the IEEE recommended limits to reduce health problems in viewers due to flicker to a low risk and to there being no observable effects when compared statistically to appropriate (flicker-free) control conditions [25]. The limits at frequencies above 90 Hz have been generalized based on PPF with limit values of 3% and 1% respectively. For the highest PPF value for school 4 only, the signal-to-noise was low; all other PPF values in school 4 were below 1%.

All the modulation of the electric lighting observed had a base frequency of 100 Hz. Three tested luminaires (19%) in the schools visited achieved the no observable effect limit or better, and one more (6%) met the less-stringent IEEE low-risk limit, see Figure 5. The signal-to-noise ratio was sufficient for the assessment in all but one measurement, where it was marginal, and this is included here, thereby materially extending the upper range limit for school 4. The mean PPF was 7.5% (s.d. 5.5%, range 0.3% to 17.0%), the mean flicker index was 7.0% (s.d. 4.7%, range 0.6% to 14.7%), and the mean unsmoothed modulation depth was 38% (s.d. 23%, range 5% to 78%). The Pearson correlation coefficients for PPF with the flicker index and modulation depth, as assessments for fixed frequency TLM, were 94% and 76%, respectively. The strong correlation with the flicker index reflects the underlying mathematical similarities of the approaches when the base frequency is constrained; the slightly less strong correlation with percent flicker may indicate a limited range of duty cycles in this sample, which was not dimmable, as installed.

## 4. Discussion

A template of school hours of 08:30 to 16:29 was adopted for the purposes of daylight analysis, although actual school time may vary and children may arrive and leave earlier or later, depending on a number of factors. This template allows for hourly subdivisions and averaging. Our time series began 10 days after the annual forward change in the clocks for daylight saving, and the light levels were lower towards the start rather than the end of the assumed school hours.

Recent published data for the seated eye height of children were not found, but a previous standard, BS EN 1729, for chair and desk heights by age were available [30]. Combined with data on Dutch children’s sitting height, these indicate 1.0 m to be a reasonable seated eye-height estimate for a typical UK 10-year-old [31]. Children’s typical standing eye-height might be around 1.3 m based on WHO-UK charts for 1990 [32], and the 1.5 m light logger position is intended to be above typical obstructions to daylight penetration in respect to classrooms and within the range of typical window heights.

Implementing and designing a rapid assessment protocol for classrooms relies on in situ decision making, and care must, therefore, be taken to outline a skeleton protocol that is robust for collecting relevant and consistent data between different classrooms. The protocol choices were informed by concurrent work on architectural lighting design of classrooms and a scoping review of myopia research including objective light assessments [33]. The illuminance data were the most time consuming to collect. We hope to include these within post-occupancy assessments in future research, and they were also originally meant to form part of a project in which we were involved that also include two schools in the Netherlands [34].

The melanopic EDI daytime recommended target was not specifically designed for school children. However, various similar provisions have been included in the “WELL Building Standard”, see [35], an opt-in building certification that can be applied to schools and that has been adopted in “healthy” architectural practise. Further evidence is needed to determine how similar children’s light exposure needs for circadian rhythms are to those of day-active adults [20]. One study on myopia prevention and outdoor light in Taiwan suggested schools including longer exposure durations at higher illuminances of 1000 to 3000 lx or more outdoors, e.g., in shaded areas, if higher illuminances outdoors could not be used [8].

Most of the classrooms visited did not provide the recommended level for daytime exposure of day-active adults. The spectra of the electric lighting reduced its contribution to these requirements, compared to the visual illumination provided, by over 50%.

An important limitation of the protocol studied is that the weather influences the assessments, particularly those during the visits. Night visits would allow for improved electric lighting assessment, and, similarly, longer sensor collection periods over different parts of the school year and during classroom occupancy would support improved daylighting assessments. A strength of the study is that it considers the possibility of different mechanisms, and the protocol is designed to collect re-analyzable spectral and temporal data.

Most of the lighting in the classroom visited modulated at 100 Hz, at levels considered above the low-risk categorization of the international recommendations. Although the possibility of a link to myopia has not been investigated epidemiologically, to our knowledge, it may impair visual performance, including near work [36], and cause headaches [37].

The PPF was chosen for assessing the electric lighting against the health recommendations of IEEE [25]. A recent proposed metric, the Phantom Array Visibility Measure (PAVM), is based on new data that could potentially describe the health-related responses considered here relative to a range of frequencies above 90 Hz [38]. An alternative metric, the Stroboscopic Visibility Measure (SVM), used in regulations in the UK only applies to new lighting [39] and is not intended to be used as a health-related measure or to predict the phantom array affect [40]. PAVM and SVM are both visibility measures, or metrics, that aggregate Fast Fourier Transform-derived amplitudes using relative frequency weightings according to human sensitivity data. In the case of PAVM, the phantom array relates to the creation of multiple images on the retina during the saccade of an eye, and for SVM, the stroboscopic effect of interest is the effect of multiple images on the retina during the rapid motion of a viewed object or stroboscopic light. Lastly, at 100 Hz, the flicker index is known to be a good indicator of temporal light artefacts in sensitive observers, and the correlation with PPF in our data reflects this [24].

## 5. Conclusions

In relation to children’s exposures in classrooms, daylight levels at eye height are variable and depend on multiple factors such as glazing area and height; room aspect, depth, and height; and weather variations and the changing position of the sun.

We consider this to be a pilot study, so the conclusions relate to the use of the protocol in the design of further research, and the findings are further supported by related modelling work [34]. While it was confirmed that post-occupancy lighting assessment of classrooms can be carried out in a thirty-minute protocol, spot illuminance measures and assessments of varied light exposure intensity are heavily weather dependent.

In addition to classroom daylighting design, interventions targeting eye-level illuminance and the spectral range, m-DER, or stability (i.e., a lack of unwanted TLM) of electric lighting may show promise for improving outcomes related to healthy regulation of circadian rhythms and eye growth in schoolchildren.

## Data Availability

The data presented in this study are available on request from the corresponding author due to the participation of third parties (schools).
